# Antinociceptive and Anti-Inflammatory Activities of the Sesame Oil and Sesamin

**DOI:** 10.3390/nu6051931

**Published:** 2014-05-12

**Authors:** Érika Maria Henriques Monteiro, Lucas Apolinário Chibli, Célia Hitomi Yamamoto, Mônica Cecília Santana Pereira, Fernanda Maria Pinto Vilela, Mírian Pereira Rodarte, Míriam Aparecida de Oliveira Pinto, Maria da Penha Henriques do Amaral, Marcelo Silva Silvério, Ana Lúcia Santos de Matos Araújo, Aílson da Luz André de Araújo, Glauciemar Del-Vechio-Vieira, Orlando Vieira de Sousa

**Affiliations:** 1 Pharmaceutical Sciences Post-Graduation Program, Faculty of Pharmacy, Federal University of Juiz de Fora, Campus Universitário, Juiz de Fora, Minas Gerais 36036-900, Brazil; E-Mails: erikahenriques07@yahoo.com.br (E.M.H.M.); lucano.farm@gmail.com (L.A.C.); 2 Department of Pharmaceutical Sciences, Faculty of Pharmacy, Federal University of Juiz de Fora, Campus Universitário, Juiz de Fora, Minas Gerais 36036-900, Brazil; E-Mails: hytomani@yahoo.com (C.H.Y.); monicasantanapereira@bol.com.br (M.C.S.P.); fernandampvilela@gmail.com (F.M.P.V.); mirianpereira.rodarte@ufjf.edu.br (M.P.R.); miriamaop@yahoo.com.br (M.A.O.P.); penhaufjf1@yahoo.com.br (M.P.H.A.); marcelosilverio@hotmail.com (M.S.S.); alsma@ig.com.br (A.L.S.M.A.); ailson.luz@ufjf.edu.br (A.L.A.A.); glauciemar@gmail.com (G.D.-V.-V.)

**Keywords:** sesame oil, sesamin, antinociceptive activity, anti-inflammatory activity

## Abstract

Sesame oil is widely consumed as nutritious food, cooking oil, and in pharmaceuticals and food. In this study, the antinociceptive and anti-inflammatory properties of the sesame oil and sesamin were investigated. The sesame oil and sesamin reduced the number of abdominal contortions at the doses 100, 200, or 400 mg/kg. The first and second phases of the time paw licking were inhibited by sesame oil and sesamin (100, 200, or 400 mg/kg). After 90 min of treatment, sesame oil and sesamin increased the reaction time on a hot plate (200 or 400 mg/kg). Considering the tail-immersion assay, the sesame oil and sesamin produced significant effect after 60 min at the doses of 100, 200, or 400 mg/kg. After 4 h of application of the carrageenan, the sesame oil and sesamin were effective against the paw edema. The exudate volume and leucocyte migration were also reduced by sesame oil and sesamin. These results suggest that sesamin is one of the active compounds found in sesame oil and justify the antinociceptive and anti-inflammatory properties of this product.

## 1. Introduction

Among the natural constituents that have revealed antinociceptive and anti-inflammatory activities are reported compounds belonging to the fixed oils and lignans [1,2]. For example, linolenic acids from Ocimum sanctum possess anti-inflammatory activity against PGE2, leukotriene and arachidonic blocking both the cyclooxygenase and lipoxygenase pathways [1,3,4,5]. The oil of the *Euterpe oleracea*, with major compounds palmitic acid, palmitoleic acid and oleic acid [6], and palmitic acid derivatives [7], showed antinociceptive and anti-inflammatory effects. Lignans, such as lariciresinol, taxiresinol, 3′-demethylisolariciresinol-9′-hydroxyisopropylether, isolariciresinol, and 3-demethylisolariciresinol isolated from *Taxus baccata* [2], as well as (+)-eudesmin, (+)-magnolin, (+)-yangambin, and epimagnolin B isolated from *Magnolia fargesii* [8] and phylligenin of *Forsythia koreana* [9] were able to inhibit inflammation and pain mechanisms in different biological models. Cubebin, a dibenzylbutyrolactone lignan isolated from the crude hexane extract of the leaves of *Zanthoxyllum naranjillo*, showed a significant anti-inflammatory activity by using the paw edema induced by carrageenan in rats [10]. In addition, arctigenin, a lignan from *Arctium lappa*, suppressed the overproduction of NO through down-regulation of iNOS expression and iNOS enzymatic activity in lipopolysaccharide LPS-stimulated macrophage [11].

Sesame oil, extracted of *Sesamum indicum* L. seeds, has been employed in the food and pharmaceutical industries due to the high lipids and protein content and its distinctive flavor [12,13]. In addition to being used as an emulsifier, the oil is rich in unsaturated fatty acids (palmitic, stearic, oleic, and linoleic acids) [14] and has anti-cholesterol property [15]. It is widely consumed as nutritious food, very beneficial to health, as cooking oil, in pharmaceuticals, in shortening and margarine, as soap fat and as synergist for insecticides [16,17]. From the viewpoint of biological activities, the presence of the natural antioxidants such as sesamol, sesamolin, and gammatocopherol gives a high oxidative stability to the sesame oil preventing to oxidation followed by free radical mechanisms [12,13,14,15,16,17,18,19]. Furthermore, the sesame oil administrated as dietary supplement produced analgesic, antipyretic and anti-inflammatory activities in experimental animal models [20].

Among the chemical constituents found in the sesame seeds, the lignans, as sesamin, asarinin, and sesamolin, play an important role in health-promoting effects [21,22]. Sesamin and sesamolin, for example, have shown antioxidant [23], antiproliferative [24,25], antihypertensive [26,27], and neuroprotective activities [28], as well as lowering cholesterol levels [29] and increasing hepatic fatty acid oxidation enzymes [30]. In addition to lignans, other bioactive compounds, such as vitamin E, have been highlighted [31,32].

Considering the applications in the food and pharmaceutical industries and the presence of antioxidants, as well as the lack of chemical evidence that justify the analgesic and anti-inflammatory properties, the present investigation was designed to evaluate the antinociceptive and anti-inflammatory activities of the sesame oil and sesamin.

## 2. Experimental Section

### 2.1. Chemicals

Drugs and reagents employed were as follows: sesame oil was purchased from local market (Hong Kong, China), sesamin (Sigma-Aldrich, St. Louis, MO, USA), acetic acid (Vetec Química Farm. Ltda, Rio de Janeiro, Brazil), formaldehyde and acetylsalicylic acid (Reagen Quimibrás Ind. Química S.A., Rio de Janeiro, Brazil), morphine hydrochloride (Merck Inc., Whitehouse Station, NJ, USA), naloxone, indomethacin, and carrageenan (Sigma Chemical Co, St. Louis, MO, USA).

### 2.2. Animals

Male Wistar rats (90–110 days) weighing 180–220 g and male Swiss albino mice (50–70 days) weighing 25–30 g were obtained from Central Biotery of the Federal University of Juiz de Fora. The animals were housed in in plastic cages (47 × 34 × 18 cm^3^) under a 12 h light/12 h dark cycle at room temperature (22 ± 2 °C) and had free Access to water and food. Animal care and the experimental protocol were in accordance to the principles and guidelines recommended by the Brazilian College of Animal Experimentation (COBEA) and were approved by the local Ethical Committee (protocol number 049/2012).

### 2.3. Acute Toxicity

Groups of ten mice received orally doses of 0.5, 1, 1.5, 2, and 3 g/kg of the oil from sesame seeds, while the control group was administered with the vehicle (saline). The groups were observed for 48 h and at the end of this period the mortality was recorded for each group [33]. The 50% lethal dose (LD_50_) was determined by probit test using a percentage of death *versus* doses’ log [34].

### 2.4. Acetic Acid-Induced Writhing Response in Mice

The acetic-acid writhing test was used for the evaluation of analgesic activity [35]. Mice (*n* = 8 per group) were injected (i.p.) with 0.6% acetic acid (10 mL/kg body weight), and the intensity of nociception was quantified by counting of the total number of writhes that occurred between 10 and 30 min after injection. Animals received the sesame oil (100, 200, and 400 mg/kg, per oral-p.o.), sesamin (50, 100 and 200 mg/kg, p.o.) and sterile saline in 1% DMSO (control group, 10 mL/kg, p.o.) 60 min before acetic acid injection. Acetylsalicylic acid (200 mg/kg, p.o.) and indomethacin (10 mg/kg, p.o.) were administered 60 min before acetic acid as reference drugs.

### 2.5. Formalin-Induced Nociception in Mice

Groups of mice (8 animals per group) were injected with 20 µL of 2.5% formalin (in 0.9% saline, subplantar) and the duration of paw licking was determined 0–5 min (first phase) and 15–30 min (second phase) after formalin injection [36]. The animals were pre-treated with the sesame oil (100, 200, and 400 mg/kg, p.o.), sesamin (50, 100, and 200 mg/kg, p.o.) and morphine (5 mg/kg, subcutaneous-s.c.) 60 min before formalin administration. Control animals were treated with similar volume of sterile saline in 1% DMSO (10 mL/kg, p.o.). Morphine (5 mg/kg, s.c.) was used as reference drug.

### 2.6. Hot Plate Latency Assay in Mice

To start this experiment, groups of eight mice (*n* = 8) were treated with the sesame oil (100, 200 and 400 mg/kg, p.o.), sesamin (50, 100, and 200 mg/kg, p.o.) or morphine (5 mg/kg, s.c.) and the control group received sterile saline in 1% DMSO (10 mL/kg, p.o.). After drug administration, the animals were placed on a Hot-Plate (Model LE 7406, Letica Scientific Instruments, Barcelona, Spain) heated at 55 ± 1 °C [37] and the latency time was measured at 0, 30, 60 and 90 min with a cut-off time of 30 s to avoid animal paw lesion. In separate groups of animals (*n* = 8), the effect of pre-treatment with naloxone (2 mg/kg, s.c.) on the analgesia also produced by the sesame oil (400 mg/kg, p.o.), sesamin (200 mg/kg, p.o.), and morphine (5 mg/kg, s.c.) was also determined.

### 2.7. Tail Immersion Test in Mice

The mice were divided into six groups of eight animals for the tail immersion that was performed according Ramabadran *et al.* (1989) [38]. The reaction time was recorded by observing tail flick response when tail is immersed in water maintained at constant temperature (55 ± 1 °C). A cut-off period of 10 s is observed to avoid tissue damage. Sterile saline in 1% DMSO (negative control, p.o.), sesame oil (100, 200 and 400 mg/kg, p.o.), sesamin (50, 100 and 200 mg/kg, p.o.) and morphine (positive control, 5 mg/kg, s.c.) were administered. The reaction time of animals was recorded at 0, 30, 60, 90, and 120 min after the drug administration.

### 2.8. Carrageenan-Induced Edema in Rats

Anti-inflammatory activity was assessed on the basis of paw edema inhibition induced by the injection of 0.1 mL 1% carrageenan (an edematogenic agent) into the subplantar region of the right hind paw of the rat [39]. Male Wistar rats were divided into groups of six animals, which received oral doses of the sesame oil (100, 200 and 400 mg/kg, p.o.), sesamin (50, 100 and 200 mg/kg, p.o.), sterile saline in 1% DMSO (p.o.) or indomethacin (10 mg/kg, p.o.) 60 min before the injection of carrageenan. In the left paw, used as a control, 0.1 mL of sterile saline was injected. 1, 2, 3, and 4 h after the carrageenan injection, the measure of edema was made by the difference between the volume displaced by the right and the left paw using a plethysmometer (model LE 7500, Letica Scientific Instruments, Barcelona, Spain).

### 2.9. Carrageenan-Induced Pleurisy in Rats

Pleurisy was induced in male Wistar rats by intrapleural administration of 0.5 mL 2% carrageenan suspension in sterile saline between the third and fifth ribs on the right side of the mediastinum [40]. Sesame oil (100, 200, and 400 mg/kg, p.o.), sesamin (50, 100, and 200 mg/kg, p.o.), sterile saline in 1% DMSO (p.o.) or indomethacin (10 mg/kg, p.o.) were given 60 min before injection of the irritant. Animals were killed 4 h after carrageenan injection, and the skin and pectoral muscles were retracted. A longitudinal incision was made between the third and fifth ribs on each side of the mediastinum. The exudate was collected and transferred to a 15 mL conical centrifuge tube and the total volume was determined. A 20 µL aliquot of the exudate was used to determine the total leucocytes using Neubauer chamber under microscopy analysis.

### 2.10. Statistical Analysis

Data are expressed as mean ± S.E.M. Statistical significance was analyzed by the one-way analysis of variance (ANOVA) followed by the Student Newman-Keuls test. *p* values below 0.05 were considered significant. The percentage of inhibition was calculated by using:

100 − T × 100/C(%) or T × 100/C − 100(%)

where C and T indicate non-treated (vehicle) and drug-treated, respectively.

## 3. Results

### 3.1. Acute Toxicity

After 48 h of treatment, the oil was not toxic to animals at the doses administered. General signs of toxicity, such as cyanosis, piloerection, writhing, ptosis, tremors, convulsions, ataxia, hypnosis, red urine, and diarrhea, were not detected. The parameters motor activity, as respiration, corneal reflex, righting and withdrawal, body tone and amount of pats, were also unaffected.

### 3.2. Acetic Acid-Induced Writhing Response in Mice

**Figure 1:**
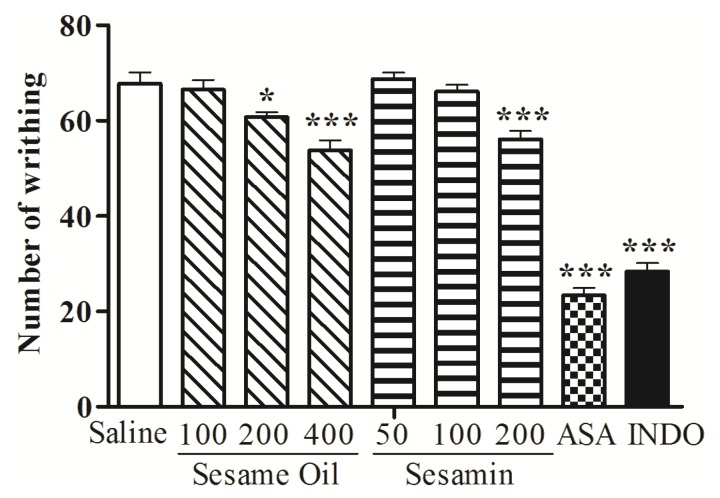
Effects of the sesame oil and sesamin on acetic acid-induced writhing in mice. ASA, acetylsalicylic acid; INDO, indomethacin. Data are mean ± S.E.M. of 8 mice. * *p* < 0.05; *** *p* < 0.001 *vs.* control group.

The treatment of animals with sesame oil (200 and 400 mg/kg, p.o.) produced a significant (*p* < 0.05 and *p* < 0.001, respectively) and dose-dependent inhibition of the abdominal writhes induced by acetic acid when compared with control (67.75 ± 2.37) (Figure 1). Sesamin at the doses of 200 mg/kg caused 17.17% (*p* < 0.001) inhibition against acetic-acid-induced abdominal writhing (Figure 1). The abdominal contortions were reduced 65.50% and 58.12% by acetylsalicylic acid and indomethacin, respectively.

### 3.3. Effects on the Nociception Induced by Formalin in Mice

**Figure 2:**
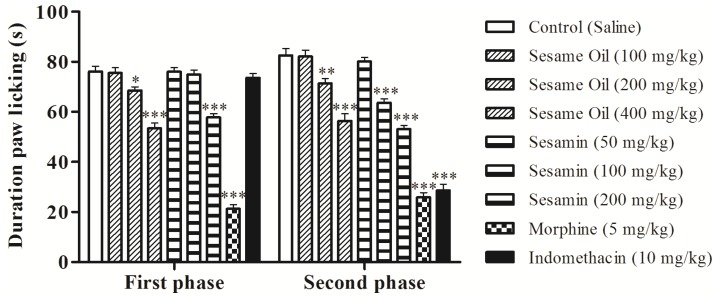
Effects of the sesame oil and sesamin on formalin-induced nociception in mice. Data are mean ± S.E.M. of 8 mice. * *p* < 0.05; ** *p* < 0.01; *** *p* < 0.001 *vs*. control group.

The intraplantar injection of formalin promoted a biphasic characteristic response (Figure 2). The time spent licking in the first phase (0–5 min) was 86.25 ± 1.98 s and in the second phase (15–30 min) was 91.62 ± 1.89 s for the control group. After 60 min of treatment, a significant reduction in the licking time (*p* < 0.05 or *p* < 0.001) was observed during the first phase (neurogenic) by 10.01%, and 29.90% with 200 and 400 mg/kg of oil, respectively (Figure 2). In this phase, sesamin reduced the paw licking time by 24.13% at the dose of 200 mg/kg (*p* < 0.001). In the second phase, the doses of 200 and 400 mg/kg of oil also inhibited significantly (*p* < 0.01 or *p* < 0.001) at 13.50% and 31.67%, respectively, when compared to the control. In addition, sesamin produced a reduction of the second phase at 22.88% (100 mg/kg) and 35.76% (200 mg/kg). As expected, morphine (5 mg/kg, s.c.) significantly reduced the formalin response in both phases. However, indomethacin was active only in the second phase.

### 3.4. Effects on Hot-Plate Latency Assay in Mice

**Figure 3:**
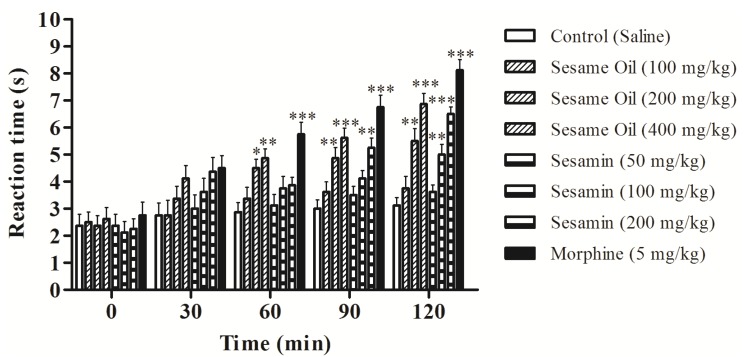
Effects of the sesame oil and sesamin on the latency time of mice exposed to the hot plate test. Data are mean ± S.E.M. of 8 mice. * *p* < 0.05; ** *p* < 0.01; *** *P* < 0.001 *vs.* control group.

Considering the result observed in the first phase of the formalin test, we decided to evaluate the oil using hot plate method, an important assay of central antinociceptive activity investigation. The effect of the oil from *S. indicum* and sesamin in the hot plate test varied according to the doses and the time of observation (Figure 3). At times 0 and 30 min, no significant antinociceptive effect was observed, while at time 60 min, the doses of 200 and 400 mg/kg of the oil and 200 mg/kg of sesamin increased significantly the latency time. After 90 min of treatment, the doses 200 and 400 mg/kg of the oil (23.96% and 55.47%; *p* < 0.01 and *p* < 0.001, respectively) and 200 mg/kg of sesamin (31.80%; *p* < 0.001) increased the reaction time. The procedure was also performed in the presence of naloxone, an opioid antagonist. It was observed that the naloxone was able to inhibit the antinociceptive effects of sesamin and morphine, but was not able to inhibit the effect of the *S. indicum* oil.

### 3.5. Effects on Tail Immersion Test in Mice

Table 1 shows the effect of the oil and sesamin on the latency of tail withdrawal from hot water. Doses of 200 (*p* < 0.05 and *p* < 0.01) and 400 mg/kg (*p* < 0.01 and *p* < 0.001) of sesame oil increased significantly the latency time on the tail-immersion in hot water after 60 and 90 min of treatment. In this last time, sesamin (200 mg/kg, *p* < 0.001) also increased the reaction time. The maximum effect was observed after 120 min of treatment with sesame oil and sesamin (*p* < 0.01 or *p* < 0.001).

**Table 1:** Effects of the sesame oil and sesamin on tail-immersion test in mice.

Group	Dose (mg/kg)	Reaction time (s)				
		0′	30′	60′	90′	120′
Control	Saline	2.37 ± 0.42	2.75 ± 0.45	2.87 ± 0.35	3.00 ± 0.33	3.12 ± 0.29
	100	2.50 ± 0.38	2.75 ± 0.56	3.37 ± 0.42	3.62 ± 0.37	3.75 ± 0.45
Oil	200	2.37 ± 0.37	3.37 ± 0.46	4.50 ± 0.33 *	4.87 ± 0.40 **	5.50 ± 0.46 **
	400	2.62 ± 0.42	4.12 ± 0.48	4.87 ± 0.35 **	5.62 ± 0.37 ***	6.87 ± 0.40 ***
	50	2.37 ± 0.42	3.00 ± 0.50	3.12 ± 0.40	3.50 ± 0.33	3.62 ± 0.26
Sesamin	100	2.12 ± 0.40	3.62 ± 0.50	3.75 ± 0.45	4.12 ± 0.29	5.00 ± 0.38 **
	200	2.25 ± 0.37	4.37 ± 0.53	3.87 ± 0.29	5.25 ± 0.37 ***	6.50 ± 0.27 ***
Morphine	5	2.75 ± 0.49	4.50 ± 0.46	5.75 ± 0.45 ***	6.75 ± 0.45 ***	8.12 ± 0.40 ***

Data are mean ± S.E.M. of 8 mice. * *p* < 0.05; ** *p* < 0.01; *** *p* < 0.001 *vs.* control group.

### 3.6. Effects on Edema Induced by Carrageenan in Rats

**Figure 4:**
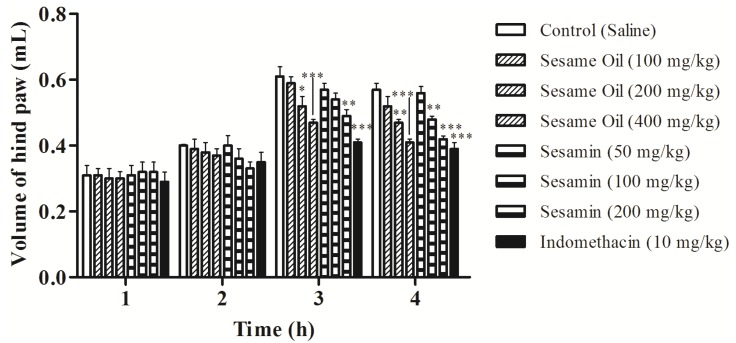
Effects of the sesame oil and sesamin on the rat paw edema induced by carrageenan. Data are mean ± S.E.M. of 6 rats. * *p* < 0.05; ** *p* < 0.01; *** *p* < 0.001 *vs.* control group.

The anti-inflammatory effect of the sesame oil and sesamin evaluated by the paw edema method induced by carrageenan is shown in Figure 4. After 3 h of carrageenan application, the paw edema was reduced in 14.75 and 22.95% at the doses of 200 and 400 mg/kg of the sesame oil, while sesamin demonstrated significant effect at the dose 200 mg/kg (19.67%). The inhibition of edema was also observed 4 h after injection of carrageenan at the doses of 200 mg/kg (17.54%; *p* < 0.01) and 400 mg/kg (28.07%; *p* < 0.001) of the sesame oil. In this time, sesamin reduced the paw edema in 15.79% and 26.31% at the doses of 100 and 200 mg/kg, respectively, when compared with control group. Indomethacin (reference drug) also inhibited the paw edema (*p* < 0.001) after 3 and 4 carrageenan injections (Figure 4).

### 3.7. Effects on Carrageenan-Induced Pleurisy in Rats

The anti-inflammatory effect of the oil and sesamin was confirmed by a decrease in the exudate volume and in the leucocyte migration (Table 2). The pleurisy effects demonstrated that doses of 200 (*p* < 0.01) and 400 mg/kg (*p* < 0.01) of sesame oil significantly reduced the exudate volume by 22.12% and 29.20%, respectively, while sesamin decreased 20.35% (100 mg/kg) and 26.55% (200 mg/kg). The sesame oil also caused inhibition of the number of total leucocytes at the doses of 200 (18.36%; *p* < 0.001) and 400 mg/kg (27.54%; *p* < 0.001) (Table 2). At the doses of 100 and 200 mg/kg of sesamin, the number of total leucocytes was significantly reduced by 12.55% (*p* < 0.01) and 23.26% (*p* < 0.001), respectively. As expected, indomethacin reduced the exudate volume and the leucocyte migration.

**Table 2:** Effects of the sesame oil and sesamin on pleural exudation and number of leucocytes in carrageenan-induced pleurisy in rats.

Group	Dose (mg/kg)	Exudate volume (mL)	Inhibition (%)	N° Leucocytes (×10^3^ cells/mm^3^)	Inhibition (%)
Control	Saline	1.13 ± 0.04	-	13.07 ± 0.31	-
	100	1.07 ± 0.03	5.31	13.03 ± 0.36	-
Sesame Oil	200	0.88 ± 0.05 **	22.12	10.67 ± 0.24 ***	18.36
	400	0.80 ± 0.06 ***	29.20	9.47 ± 0.44 ***	27.54
	50	1.02 ± 0.04	11.50	13.00 ± 0.27	-
Sesamin	100	0.90 ± 0.04 **	26.55	11.43 ± 0.25 **	12.55
	200	0.83 ± 0.03 ***	31.86	10.03 ± 0.21 ***	23.26
Indomethacin	10	0.72 ± 0.04 ***	36.28	8.43 ± 0.30 ***	35.50

Data are mean ± S.E.M. of six rats. ** *p* < 0.01, *** *p* < 0.001 *vs.* control group.

## 4. Discussion

Considering that the use of analgesic and anti-inflammatory drugs exerts a wide range of side effects [41], there is currently a strong interest in developing new therapeutic agents from natural products [42]. These products can inhibit mediators that are involved in the evolution of inflammatory processes, including the pain [43]. In this context, studies have been carried out with natural products in models of pain and inflammation in order to assess their pharmacological potential, as well as developing new therapeutic options [42]. For these reasons, we evaluated the antinociceptive and anti-inflammatory properties of the sesame oil and sesamin.

The acute toxicity test on mice showed that no animal died within 48 hours after treatment with oil. In addition, the major signs of toxicity, as well as parameters motor activity, were unaffected. This finding is relevant as regards the safety of the use of sesame oil in the diet and therapeutic applications.

Intraperitoneal injection of acetic acid induces an increase of the level of prostanoids, particularly PGE_2_ and PGF_2α_, as well as lipoxygenase products in the peritoneal fluid of mice with sensitization of nociceptors [36] and generation of bradykinin, histamine, and serotonin in this fluid [44]. Our results revealed that the sesame oil and sesamin reduced the abdominal writhes induced by acetic acid showing antinociceptive effect. In addition, our data extend previous observations showing that palmitic acetic [7] and sesamin [45,46] inhibit acid-induced in mice. The mechanism of this effect could be related the prostaglandin synthesis inhibition as suggested for linoleic acid, one of the constituents of the sesame oil [3].

The formalin injection causes a local tissue injury into the paw and is associated to two distinct phases [37]. The first phase (0–5 min after formalin injection) is due to a direct effect on nociceptors and the second phase (15–30 min after formalin injection) produces an inflammatory response that involves different chemical mediators, such as excitatory amino acids, neuropeptides, PGE2, nitric oxide, and kinins [37,47]. This model has been commonly used to establish antinociceptive mechanism of analgesic drugs [48]. Centrally acting drugs such as opioids inhibit both phases equally, but peripherally acting drugs, such as aspirin, indomethacin, and dexamethasone, inhibit only the second phase [49]. We observed that sesame oil and sesamin were able to decrease the time that the animal spent licking the injected paw on first and second phases (Figure 2), confirming reports described previously [20,47,48]. Taken together, these results revealed a probably similar action to the opioid and nonsteroidal anti-inflammatory drugs.

Nociceptive reaction towards thermal stimuli in hot plate test and tail immersion in hot water test using mice is a well-validated model for the detection of opiate analgesic as well as several types of analgesic drugs from spinal origin [50]. The hot plate is a specific central antinociceptive test in which opioid agents exert their analgesic effects via supra spinal and spinal receptors [51]. The tail-immersion assay indicated that the pharmacological actions were mediated by µ opioid receptors rather than κ and δ receptors [52]. These models are also related to drugs that act on supreespinhal level through mechanisms of pain modulation [53]. Our results demonstrated that the oral administration of the sesame oil and sesamin exerted significant prolongation in the response latency time to the heat stimulus (Figure 3 and Table 1) and are in agreement with the action of fatty acids [7,54] and sesamin [46] against thermal-induced nociception. In addition, the analgesia induced by the sesame oil may be associated to the opioid system, since previous treatment with naloxone not completely changed the observed data, but blocked the effect of the sesamin (Figure 3). Therefore, sesamin produced a similar effect to morphine that significantly increased the latency time to the nociceptive response when compared with the control group.

Carrageenan-induced rat paw edema is a model that has been widely used to assess anti-inflammatory activity of natural products and consists of three phases [55]. The first phase (1–2 h) is related with the release of serotonin and histamine; kinins play a role in the middle phase, while prostaglandins appear to be the most important mediators in the second phase (3–5 h) of the postcarrageenan response as a resulted of induction of isoforms of cyclooxygenase [56]. The result of the present study indicates that the suppression of the second phase may be due to cyclooxygenase inhibition with reduced expression of prostaglandins. In this context, the sesame oil and sesamin play a crucial role as protective factors against the carrageenan-induced acute inflammation (Figure 4). This finding corroborates the anti-inflammatory activity of unsaturated fatty acids, as linoleic and linolenic acids, which significantly inhibited the edema induced by PGE2, LTB4, arachidonic acid and carrageenan [3].

To better understanding of the anti-inflammatory effect demonstrated in the paw edema model, we performed the pleurisy test with application of carrageenan into the pleural cavity of rats. This test elicits an acute inflammatory response, characterized by the accumulation of fluid containing large number of leucocytes [57]. It is an interesting method that evaluates the leucocyte migration during the inflammatory process. Anti-inflammatory drugs, such as indomethacin and dexamethasone, inhibit the accumulation of exudates and mobilization of leucocytes between 3 and 6 h after application of carrageenan [58]. Our results showed that the sesame oil and sesamin inhibited the formation of pleural exudate and the leucocyte migration confirming the anti-inflammatory activity (Table 2).

From the chemical point of view, it is known that sesame oil has a high content of unsaturated fatty acids (palmitic, stearic, oleic and linoleic acids) [14], well as well presence of lignans (sesamin, asarinin, sesamolin, and sesamol) and gamma-tocopherol [18]. These compounds may be responsible for the pharmacological activities, since several studies have indicated that fatty acids reduce the levels of prostaglandins and leukotrienes [1,2,3,4,5,6,7]. Linoleic acid, for example, could inhibit the formation of prostaglandins and leukotrienes from arachidonic acid [3], while palmitic acid and derivatives decreased the thermal nociception in mice [7]. In addition, lignans, such as sesamin, have also been described with activity against pain and inflammation [8,9,10,11,46,47]. However, further studies are necessary to understand the mechanisms of action and correlate the pharmacological activity with the chemical composition of sesame oil.

## 5. Conclusions

The results obtained through the animal model experiments performed in the present study adding more subsidies to the use of sesame oil due to their antinociceptive and anti-inflammatory properties. Based on our data, sesamin is one of the active constituents of sesame oil and represents a promising target for the treatment of pain and inflammation. However, further studies should be conducted to ensure the safety, feasibility, and sustainability of usage.

## References

[1] Singh S., Majumdar D.K. (1995). Anti-inflammatory and antipyretic activities of *Ocimum Sanctum* fixed oil. Pharm. Biol..

[2] Küpeli E., Erdemoğlu N., Yeşilada E., Sener B. (2003). Anti-inflammatory and antinociceptive activity of taxoids and lignans from the heartwood of *Taxus baccata* L.. J. Ethnopharmacol..

[3] Singh S., Taneja M., Majumdar D.K. (2007). Biological activities of *Ocimum sanctum* L. fixed oil—An overview. Indian J. Exp. Biol..

[4] Singh S., Majumdar D.K., Rehan H.M. (1996). Evaluation of anti-inflammatory potential of fixed oil of *Ocimum sanctum* (Holybasil) and its possible mechanism of action. J. Ethnopharmacol..

[5] Singh S. (1999). Mechanism of action of anti-inflammatory effect of fixed oil of *Ocimum basilicum* Linn. Indian J. Exp. Biol..

[6] Favacho H.A.S., Oliveira B.R., Santos K.C., Medeiros B.J.L., Sousa P.J.C., Perazzo F.F., Carvalho J.C.T. (2011). Anti-inflammatory and antinociceptive activities of *Euterpe oleracea* oil. Braz. J. Pharmacogn..

[7] Déciga-Campos M., Montiel-Ruiz R.M., Navarrete-Vázquez G., ópez-Muñoz F.J. (2007). Palmitic acid analogues exhibiting antinociceptive activity in mice. Proc. West. Pharmacol. Soc..

[8] Kim J.Y., Lim H.J., Lee D.Y., Kim J.S., Kim D.H., Lee H.J., Kim H.D., Jeon R., Ryu J.-H. (2009). *In vitro* anti-inflammatory activity of lignans isolated from *Magnolia fargesii*. Bioorg. Med. Chem. Lett..

[9] Lim H., Lee J.G., Lee S.H., Kim Y.S., Kim H.P. (2008). Anti-inflammatory activity of phylligenin, a lignan from the fruits of *Forsythia koreana*, and its cellular mechanism of action. J. Ethnopharmacol..

[10] Bastos J.K., Carvalho J.C., de Souza G.H., Pedrazzi A.H., Sarti S.J. (2001). Anti-inflammatory activity of cubebin, a lignan from the leaves of *Zanthoxyllum naranjillo* Griseb. J. Ethnopharmacol..

[11] Zhao F., Wang L., Liu K. (2009). *In vitro* anti-inflammatory effects of arctigenin, a lignan from *Arctium lappa* L., through inhibition on iNOS pathway. J. Ethnopharmacol..

[12] Abou-Gharbia H.A., Shahidi F., Shehata A.A.Y., Youssef M.M. (1997). Effect of processing on oxidative stability of sesame oil extracted from intact and dehulled seed. J. Am. Oil Chem. Soc..

[13] Abou-Gharbia H.A., Shehata A.A.Y., Shahidi F. (2000). Effect of processing on oxidative stability and lipid classes of sesame oil. Food Res. Int..

[14] Carvalho R.H.R., Galvão E.L., Barros J.Â.C., Conceição M.M., Sousa E.M.B.D. (2012). Extraction, fatty acid profile and antioxidant activity of sesame extract (*Sesamum Indicum* L.). Braz. J. Chem. Eng..

[15] Isha D., Milind P. (2012). Eat til and protect dil. Int. Res. J. Pharm..

[16] Xu J., Chen S., Hu Q. (2005). Antioxidant activity of brown pigment and extracts from black sesame seed (*Sesamum indicum* L). Food Chem..

[17] Doker O., Salgin U., Yieldiz N., Aydognus M., Calimi A. (2010). Extraction of sesame seed oil using supercritical CO_2_ and mathematical modeling. J. Food Eng..

[18] Corso M.P., Klen M.F., Silva E.A., Filho L.C., Santos J.N., Freitas L.S., Dariva C. (2010). Extraction of sesame seed (*Sesamun indicum* L) oil using compressed propane and supercritical carbon dioxide. J. Supercrit. Fluids.

[19] Rangkadilok N., Pholphana N., Mahidol C., Wongyai W., Saengsooksree K., Nookabkaew S., Satayavivad J. (2010). Variation of sesamin, sesamolin and tocopherols in sesame (*Sesamum indicum* L.) seeds and oil products in Thailand. Food Chem..

[20] Saleem T.S.M., Basha S.D., Mahesh G., Rani P.V.S. (2011). Analgesic, anti-pyretic and anti-inflammatory activity of dietary sesame oil in experimental animal models. Pharmacologia.

[21] Schwertner H.A., Rios D.C. (2012). Analysis of sesamin, asarinin, and sesamolin by HPLC with photodiode and fluorescent detection and by GC/MS: Application to sesame oil and serum samples. J. Am. Oil Chem. Soc..

[22] Wu W.-H. (2007). The contents of lignans in commercial sesame oils of Taiwan and their changes during heating. Food Chem..

[23] Suja K.P., Jayalekshmy A., Arumughan C. (2004). Free radical scavenging behavior of antioxidant compounds of sesame (*Sesamum indicum* L.) in DPPH˙ system. J. Agric. Food Chem..

[24] Yokota T., Matsuzaki Y., Koyama M., Hitomi T., Kawanaka M., Enoki-Konishi M., Okuyama Y., Takayasu J., Nishino H., Nishikawa A. (2007). Sesamin, a lignan of sesame, down-regulates cyclin D1 protein expression in human tumor cells. Cancer Sci..

[25] Ghafoorunissa, Hemalatha S., Rao M.V.V. (2004). Sesame lignans enhance the antioxidant activity of vitamin E in lipid peroxidation systems. Mol. Cell. Biochem..

[26] Lee C.C., Chen P.R., Lin S., Tsai S.C., Wang B.W., Chen W.W., Tsai C.E., Shyu K.G. (2004). Sesamin induces nitric oxide and decreases endothelin-1 production in HUVECs: Possible implications for its antihypertensive effect. J. Hypertens..

[27] Nakano D., Kurumazuka D., Nagai Y., Nishiyama A., Kiso Y., Matsumura Y. (2008). Dietary sesamin suppresses aortic NADPH oxidase in DOCA salt hypertensive rats. Clin. Exp. Pharmacol. Physiol..

[28] Cheng F.C., Jinn T.R., Hou R.C., Tzen J.T.C. (2006). Neuroprotective effects of sesamin and sesamolin on gerbil brain in cerebral ischemia. Int. J. Biomed. Sci..

[29] Visavadiya N.P., Narasimhacharya A.V.R.L. (2008). Sesame as a hypocholesteraemic and antioxidant dietary component. Food Chem. Toxicol..

[30] Ashakumary L., Rouyer I., Takahashi Y., Ide T., Fukuda N., Aoyama T., Hashimoto T., Mizugaki M., Sugano M. (1999). Sesamin, a sesame lignan, is a potent inducer of hepatic fatty acid oxidation in the rat. Metabolism.

[31] Williamson K.S., Morris J.B., Pye Q.N., Kamat C.D., Hensley K. (2008). A survey of sesamin and composition of tocopherol variability from seeds of eleven diverse sesame (*Sesamum indicum* L.) genotypes using HPLC-PAD-ECD. Phytochem. Anal..

[32] Hemalatha S., Ghafoorunissa (2004). Lignans and tocopherols in Indian sesame cultivars. J. Am. Oil Chem. Soc..

[33] Lorke D. (1983). A new approach to pratical acute toxicity testing. Arch. Toxicol..

[34] Litchfield J.T., Wilcoxon F. (1949). A simplified method of evaluating dose-effect experiments. J. Pharmacol. Exp. Ther..

[35] Schmidt A.P., Böhmer A.E., Schallenberger C., Antunes C., Tavares R.G., Wofchuk S.T., Elisabetsky E., Souza D.O. (2010). Mechanisms involved in the antinociception induced by systemic administration of guanosine in mice. Br. J. Pharmacol..

[36] Hunskaar S., Hole K. (1987). The formalin test in mice: dissociation between inflammatory and noninflammatory pain. Pain.

[37] Eddy N.B., Leimbach D. (1953). Synthetic analgesics. II. Dithienylbutenyl and dithienylbutilamines. J. Pharmacol. Exp. Ther..

[38] Ramabadran K., Bansinath M., Turndorf H., Puig M.M. (1989). Tail immersion test for the evaluation of a nociceptive reaction in mice: Methodological considerations. J. Pharmacol. Methods.

[39] Winter C.A., Risley E.A., Nuss G.W. (1962). Carrageenin-induced edema in hind paw of the rat as an assay for antiinflammatory drugs. Proc. Soc. Exp. Biol. Med..

[40] Vinegar R., Truax J.F., Selph J.L. (1973). Some quantitative temporal characteristics of carrageenin-induced pleurisy in the rat. Proc. Soc. Exp. Biol. Med..

[41] Fujimori S., Gudis K., Sakamoto C. (2010). A review of anti-inflammatory drug-induced gastrointestinal injury: Focus on prevention of small intestinal injury. Pharmaceuticals.

[42] Newman D.J., Cragg G.M. (2012). Natural products as sources of new drugs over the 30 years from 1981 to 2010. J. Nat. Prod..

[43] Kidd B.L., Urban L.A. (2001). Mechanisms of inflammatory pain. Br. J. Anaesth..

[44] Deraedt R., Jouquey S., Delevallée F., Flahaut M. (1980). Release of prostaglandins E and F in an algogenic reaction and its inhibition. Eur. J. Pharmacol..

[45] Guo T., Deng Y.X., Xie H., Yao C.Y., Cai C.C., Pan S.L., Wang Y.L. (2011). Antinociceptive and anti-inflammatory activities of ethyl acetate fraction from *Zanthoxylum armatum* in mice. Fitoterapia.

[46] Lima L.M., Perazzo F.F., Tavares Carvalho J.C., Bastos J.K. (2007). Anti-inflammatory and analgesic activities of the ethanolic extracts from *Zanthoxylum riedelianum* (Rutaceae) leaves and stem bark. J. Pharm. Pharmacol..

[47] Shibata M., Ohkubo T., Takahashi H., Inoki R. (1989). Modified formalin test: characteristic biphasic pain response. Pain.

[48] Tjolsen A., Berge O.G., Hunskaar S., Rosland J.H., Hole K. (1992). The formalin test: An evaluation of the method. Pain.

[49] Rosland J.H., Tjolsen A., Maehle B., Hole K. (1990). The formalin test in mice: effect of formalin concentration. Pain.

[50] Abbott F.V., Melzack R. (1982). Brainstem lesions dissociated neural mechanisms of morphine analgesia in different kinds of pain. Brain Res..

[51] Nemirovsky A., Chen L., Zelman V., Jurna I. (2011). The antinociceptive effect of the combination of spinal morphine with systemic morphine or buprenorphine. Anesth. Analg..

[52] Schmauss C., Yaksh T.L. (1984). *In vivo* studies on spinal opiate receptor systems mediating antinociception. II. Pharmacological profiles suggesting a differential association of mu, delta and kappa receptors with visceral chemical and cutaneous thermal stimuli in the rat. J. Pharmacol. Exp. Ther..

[53] Yaksh T.L., Rudy T.A. (1977). Studies on direct spinal action of narcotics in production of analgesia in rat. J. Pharmacol. Exp. Ther..

[54] Zakaria Z.A., Mat Jais A.M., Goh Y.M., Sulaiman M.R., Somchit M.N. (2007). Amino acid and fatty acid composition of an aqueous extract of *Channa striatus* (Haruan) that exhibits antinociceptive activity. Clin. Exp. Pharmacol. Physiol..

[55] Eddouks M., Chattopadhyay D., Zeggwagh N.A. (2012). Animal models as tools to investigate antidiabetic and anti-inflammatory plants. Evid.-Based Complement. Altern. Med..

[56] Posadas I., Bucci M., Roviezzo F, Rossi A., Parente L., Sautebin L., Cirino G. (2004). Carrageenan induced mouse paw oedema is biphasic, age-weight dependent and displays differential nitric oxide cyclooxygenase-2 expression. Br. J. Pharmacol..

[57] Patel M., Murugananthan G., Gowda K.P.S. (2012). *In vivo* animal models in preclinical evaluation of anti-inflammatory activity—A review. Int. J. Pharm. Res. Allied Sci..

[58] Almeida A.P., Bayer B.M., Horakova Z., Beaven M.A. (1980). Influence of indomethacin and other anti-inflammatory drugs on mobilization and production of neutrophils: Studies with carrageenan induced inflammation in rats. J. Pharmacol. Exp. Ther..

